# Diagnosis of Cardiac Amyloidosis Using a Radiomics Approach Applied to Late Gadolinium-Enhanced Cardiac Magnetic Resonance Images: A Retrospective, Multicohort, Diagnostic Study

**DOI:** 10.3389/fcvm.2022.818957

**Published:** 2022-03-30

**Authors:** Xi Yang Zhou, Chun Xiang Tang, Ying Kun Guo, Xin Wei Tao, Wen Cui Chen, Jin Zhou Guo, Gui Sheng Ren, Xiao Li, Song Luo, Jun Hao Li, Wei Wei Huang, Guang Ming Lu, Long Jiang Zhang, Xiang Hua Huang, Yi Ning Wang, Gui Fen Yang

**Affiliations:** ^1^Department of Nuclear Medicine, Jinling Hospital, Medical School of Nanjing University, Nanjing, China; ^2^Department of Diagnostic Radiology, Jinling Hospital, Medical School of Nanjing University, Nanjing, China; ^3^Department of Radiology, West China Second University Hospital, Sichuan University, Chengdu, China; ^4^Bayer Healthcare, Shanghai, China; ^5^National Clinical Research Center of Kidney Disease, Jinling Hospital, Nanjing University School of Medicine, Nanjing, China; ^6^Department of Radiology, Peking Union Medical College Hospital, Chinese Academy of Medical Sciences and Peking Union Medical College, Beijing, China

**Keywords:** cardiac magnetic resonance imaging, late gadolinium enhancement, radiomics, cardiac amyloidosis, diagnostic performance

## Abstract

**Objectives:**

To assess the potential of a radiomics approach of late gadolinium enhancement (LGE) cardiac magnetic resonance (CMR) in the diagnosis of cardiac amyloidosis (CA).

**Materials and Methods:**

This retrospective study included 200 patients with biopsy-proven light-chain (AL) amyloidosis. CA was diagnosed on the basis of systemic amyloidosis confirmed with evidence of cardiac involvement by imaging and clinical biomarkers. A total of 139 patients [54 ± 8 years, 75 (54%) men] in our institution were divided into training cohort [*n* = 97, mean age of 53 ± 8 years, 54 (56%) men] and internal validation cohort [*n* = 42, mean age: 56 ± 8 years, 21 (50%) men] with a ratio of 7:3, while 61 patients [mean age: 60 ± 9 years, 42 (69%) men] from the other two institutions were enrolled for external validation. Radiomics features were extracted from global (all short-axis images from base-to-apex) left ventricular (LV) myocardium and three different segments (basal, midventricular, and apex) on short-axis LGE images using the phase-sensitive reconstruction (PSIR) sequence. The Boruta algorithm was used to select the radiomics features. This model was built using the XGBoost algorithm. The two readers performed qualitative and semiquantitative assessment of the LGE images based on the visual LGE patterns, while the quantitative assessment was measured using a dedicated semi-automatic CMR software. The diagnostic performance of the radiomics and other qualitative and quantitative parameters were compared by a receiver operating characteristic (ROC) curve analysis. A correlation between radiomics and the degree of myocardial involvement by amyloidosis was tested.

**Results:**

A total of 1,906 radiomics features were extracted for each LV section. No statistical significance was indicated between any two slices for diagnosing CA, and the highest area under the curve (AUC) was found in basal section {0.92 [95% confidence interval (CI), 0.86–0.97] in the LGE images in the training set, 0.89 (95% CI, 0.79–1.00) in the internal validation set, and 0.92 (95% CI, 0.85–0.99) in the external validation set}, which was superior to the visual assessment and quantitative LGE parameters. Moderate correlations between global or basal radiomics scores (Rad-scores) and Mayo stage in all patients were reported (Spearman’s Rho = 0.61, 0.62; all *p* < 0.01).

**Conclusion:**

A radiomics analysis of the LGE images provides incremental information compared with the visual assessment and quantitative parameters on CMR to diagnose CA. Radiomics was moderately correlated with the severity of CA. Further studies are needed to assess the prognostic significance of radiomics in patients with CA.

## Introduction

Cardiac amyloidosis (CA) is an infiltrative disease characterized by the deposition of misfolded proteins in an extracellular space of the myocardium, resulting in heart failure, conduction system disease, and sudden death (1–3). Immunoglobulin light-chain (AL) amyloidosis is the most common type of CA. Approximately 50–70% of patients with AL amyloidosis show cardiac involvement (4). Although new chemotherapy regimens and stem cell transplantation have been associated with an improvement in the survival of patients with AL amyloidosis, cardiac involvement remains the leading cause of mortality in systemic amyloidosis (5). However, the number of patients diagnosed with CA has increased exponentially due to an improvement in diagnostic modalities over the past few years (6). Given the poor prognosis and increasing incidence of CA, early detection and precise diagnosis are critical to improve the quality of life and survival of patients with CA.

Diagnosing CA is challenging based on non-specific clinical symptoms, cardiac biomarkers, and transthoracic echocardiographic abnormalities, especially in the cases accompanied by the comorbidities that could cause myocardial hypertrophy (7, 8). Endomyocardial biopsy (EMB) is the diagnostic gold standard for CA but not widely available due to its invasive nature. Cardiac magnetic resonance (CMR) offers novel techniques for detecting and quantifying the disease burden of CA. T1 mapping could provide the quantitative measurement of amyloid deposition with a high diagnostic accuracy of CA (9), whereas the myocardium with mildly or locally elevated T1 was prone to be underestimated in an uncertain “gray zone” (10). Gadolinium-based contrast-enhanced CMR was proven to be a surrogate for EMB in detecting and quantifying the disease burden of CA, particularly late gadolinium enhancement (LGE) CMR, demonstrating excellent diagnostic accuracy compared with EMB, which is an independent predictor of mortality in AL amyloidosis (10–13). Moreover, LGE magnetic resonance imaging (MRI) can be used to identify CA qualitatively and quantitatively and is the well-established method for identifying cardiac involvement in amyloidosis. However, the diagnosis of CA using LGE images is based upon the visual assessment or quantitative measurement of amyloids, which might underestimate the prevalence of CA in those patients without any significant LGE at earlier stages of amyloid deposition in the myocardium (14). These potential limitations may be overcome with recently developed radiomics-based approaches. Radiomics is a high-throughput computational method that uses microscale quantitative data hidden within conventional imaging to provide tissue characteristics, which is imperceptible to the human eyes (15). Several studies have demonstrated the feasibility and diagnostic and prognostic values of CMR radiomics (16–20). However, the potential of a radiomics-based analysis with CMR in detecting CA remains unknown.

We hypothesize that a radiomics analysis of LGE images would provide improved diagnostic information in patients with suspected cardiac AL amyloidosis. Therefore, we aimed to assess the value of CMR-based radiomics approaches for the identification of cardiac involvement in patients with systemic AL amyloidosis focusing on conventional LGE images.

## Materials and Methods

### Patient Population

This multicenter study included 200 patients with biopsy-proven AL amyloidosis. Of them, 152 consecutive patients with AL amyloidosis were prospectively collected at Jinling Hospital, Medical School of Nanjing University, China. All 152 patients received CMR imaging between November 2016 and October 2020. Inclusion criteria were patients with biopsy-proven AL amyloidosis with positive Congo red staining and AL deposition. Exclusion criteria were contraindications to CMR, including pacemakers, severe claustrophobia, implantable cardioverter–defibrillators, or the estimated glomerular filtration rate (eGFR) < 30 ml/min/1.73 m^2^, patients after autologous hematopoietic cell transplantation at admission, non-diagnostic LGE images, unsuitable for radiomics feature extraction, and image loss. To further assess the model performance and generalizability, we retrospectively collected 62 patients from the other two institutes, including Peking Union Medical College Hospital, Beijing, China and the Second West China Hospital, Sichuan University, Chengdu, China.

Cardiac amyloidosis was managed and diagnosed based on the current guidelines (21, 22). Cardiac involvement was confirmed with at least one of the following items: (1) EMB demonstrates amyloid deposits after Congo red staining and (2) positive extracardiac biopsy accompanied either by echocardiographic evidence of left ventricular (LV) wall thickening ≥ 12 mm in the absence of any other known causes, or N-terminal pro-B-type natriuretic peptide (NT-proBNP) > 332 ng/L in the absence of renal insufficiency and atrial fibrillation. All patients underwent laboratory examination of the cardiac biomarkers Troponin T (cTnT) and NT-proBNP, serum immunoglobulin-free light-chain difference (dFLC) at baseline. The severity of CA was classified into four stages (Mayo stages I, II, III, or IV) based on these biomarkers according to the revised Mayo stage published in 2012 (23).

This study protocol was approved by local institutional research ethics committees and in compliance with the Declaration of Helsinki. All patients in Jinling Hospital gave written informed consent. No written informed consent was required from the other two institutes because of the retrospective *post hoc* analysis of their clinical routine data.

### Cardiac Magnetic Resonance Imaging

#### Acquisition Protocols

All CMRs in Jinling Hospital were performed using a 3.0T scanner (TIM Trio, Siemens, Germany), while a 3T whole-body scanner (MAGNETOM Skyra, Siemens Healthineers, Erlangen, Germany) was used in both Peking Union Medical College Hospital and the West China Second University Hospital. Two-dimensional (2D) scout images were first acquired in axial, coronal, and sagittal views for the localization of the heart. Cine images on short-axis views, two-, and four-chamber long-axis views were obtained by a steady-state free precession sequence during end-expiratory breath holding. The key parameters were as follows: repetition time (TR), 50.9 ms; echo time (TE), 1.5 ms; flip angle (FA), 50°; voxel size, 1.9 mm × 1.3 mm × 8.0 mm; and slice thickness, 8 mm. LGE images were acquired about 8 min after intravenous administration of 14–18 ml of gadolinium-based contrast agent (Gadovist, Bayer Schering Health Care Limited, Reading, United Kingdom) using the inversion recovery method with phase-sensitive reconstruction (PSIR) in the views identical to the cine sequence. A dedicated inversion time (TI) scouting sequence was used before the acquisition of LGE images to adjust the optimal TI. Imaging parameters were as follows: TR = 636 ms, TE = 1.4 ms, FOV = 360 mm × 360 mm, FA = 50°, voxel size = 2.5 mm × 1.9 mm × 5.0 mm, and section thickness = 5 mm. CMR sequence and protocol details of the two external cohorts are seen in Supplementary Appendix.

#### Image Analysis

Two experienced cardiovascular radiologists (XYZ and WWH with 3 and 5 years of experience in CMR interpretation, respectively) in our core lab independently analyzed the LGE patterns of CA by visual assessment based on previous reports (24, 25), blinded to the clinical information, and laboratory tests. A CMR-based diagnosis of CA was supposed if a characteristic LGE pattern indicative of CA (comprising all of the following criteria) was observed: (1) subendocardial to transmural LGE pattern predominantly in the basal LV segments; (2) no LGE distribution correlating to the perfusion area of a coronary artery and suggesting an ischemic myocardial scar; and (3) no sharp demarcation and rather a diffuse and an extensive LGE pattern (26). Any discrepancies between the two readers were adjudicated by a senior observer (SL, with 12 years of experience in CMR interpretation). The extent of LGE was also evaluated simultaneously using the Query Amyloid Late Enhancement (QALE) score as previously reported, which semi-quantified LGE in both LV and right ventricular (RV) myocardia (27). The QALE score was assessed on the short axial LGE images at the base, mid-ventricle, and apex slices. The maximal QALE score of LV at each slice is 4 (0, absent; 1, non-specific or non-circumferential subendocardial; 2, circumferential subendocardial; 3, non-circumferential transmural; and 4, circumferential transmural), corresponding to the total maximum LGE score of 12 in 3 slices, plus 6 if LGE is present in the RV myocardium. The range of semiquantitative QALE score for the whole heart in each patient is from 0 (no detectable LGE in the LV or RV myocardium) to 18 (global transmural LGE with the involvement of both LV and RV) (24, 27).

Cardiac function analysis and LGE quantitation were performed semi-automatically with a dedicated CMR software cvi42 (version 5.13.5, Circle Cardiovascular Imaging, Inc., Calgary, AB, Canada). Structural and functional parameters were analyzed based on short-axis cine images at the end-systolic and end-diastolic cardiac phases. For LGE quantitation, epicardial and endocardial contours of LV were manually traced in short-axis LGE images. User-defined referral regions were drawn in the normal myocardium, and the regions of LGE were defined when the signal intensity exceeded 5 standard deviations (SDs) of referral regions (28). The regions of LGE were fine-tuned by the operator to reduce false positives when necessary. Values of quantitative measurement of LGE were reported as a percentage of the LV enhanced area, the total enhanced mass, and the total enhanced volume. The same software and measurements were used in all patients and controls in our study.

### Radiomics Analysis

#### Myocardial Segmentation

Free-hand regions of interest (ROIs) of the myocardium were the area between the endocardium and epicardium produced by contours from the basal to the apical slices of the short axial LV myocardium based on a scientific research platform^1^. This online platform has been developed based on the PyRadiomics library^2^. The contours were drawn carefully to avoid the involvement of the trabeculated layer and epicardial boundary. For inter-observer reproducibility, a second operator (JHL, with 2 years of experience in CMR) re-segmented independently 60 randomly selected cases, of which 2 patients failed in radiomics feature extraction, and 58 cases were finally analyzed for reproducibility.

#### Features Extraction, Selection, and Radiomics Signature Construction

Radiomics features of the three sections (basal, mid, and apical segments of the LV) and global LV (from base-to-apex) were extracted on short-axis LGE images using the PSIR sequence, respectively. Radiomics is engaged with the signal intensity measurement in an LGE sequence. All radiomics features were extracted from the scientific research platform (see text footnote 1). Then, A total of 1,906 radiomics features were then extracted for each section. For images with different slice thicknesses, the interpolator used for resampling was B-spline interpolation. For the discretization of the image gray levels, the bin width was set as 5 for MRI.

To reduce the dimensionality in our feature selection data set, the inter-observer reproducibility of all the extracted texture features was evaluated by calculating intra-class correlation coefficient (ICC), and those of ICC less than 0.80 were excluded. The remaining radiomics features were selected using the Boruta algorithm. The XGBoost algorithm was used to select the most useful radiomics features from the training cohort. In total, the extracted radiomics features were grouped as follows: (1) 396 first-order features, (2) 14 shape features, (3) 484 gray-level co-occurrence matrix (GLCM) features, (4) 352 gray-level size zone matrix (GLSZM) features, (5) 352 gray-level run-length matrix (GLRLM) features, and (6) 308 gray-level dependence matrix (GLDM) features. In total, 1,906 radiomics features were extracted, including 100 from the original images, 344 from the LoG-filtered images, 688 from the wavelet-transformed images, 344 from the local binary pattern images, and 430 [86 × 5] from non-linear intensity transforms. For each patient, the XGBoost algorithm computes an individualized radiomics score (Rad-score) according to the weighting of each variable. The predictive accuracy of the Rad-score was evaluated by the area under the curve (AUC) in both the training and validation cohorts.

### Statistical Analysis

Statistical analysis was performed using SPSS Statistics version 26.0 (IBM SPSS Inc.), R software version 4.0.2^3^, and MedCalc software version 19.6 (MedCalc Software). Based on the distribution, data are presented as mean ± SD or median and 25th–75th percentiles for continuous data. Differences between continuous data were tested using an unpaired *t*-test or the Mann–Whitney rank sum test for the two groups. Categorical variables were described as number (%) and compared using the χ^2^ or the Fisher’s exact test, as appropriate. Youden index was used to determine optimal cutoff values for cardiac involvement. The diagnostic performance of optimal cutoff values on radiomics includes accuracy, sensitivity, specificity, positive predictive value (PPV), negative predictive value (NPV), and AUC with 95% confidence intervals (CIs) to identify CA. Receiver operating characteristic (ROC) curves were compared according to the DeLong method. Inter-observer variabilities were examined for a qualitative LGE pattern and the semiquantitative QALE score using the ICC. Correlations between the parameters were analyzed using the Spearman’s correlation analysis. A two-tailed value of *p* < 0.05 was regarded to indicate statistical significance.

## Results

### Patients

**FIGURE 1:**
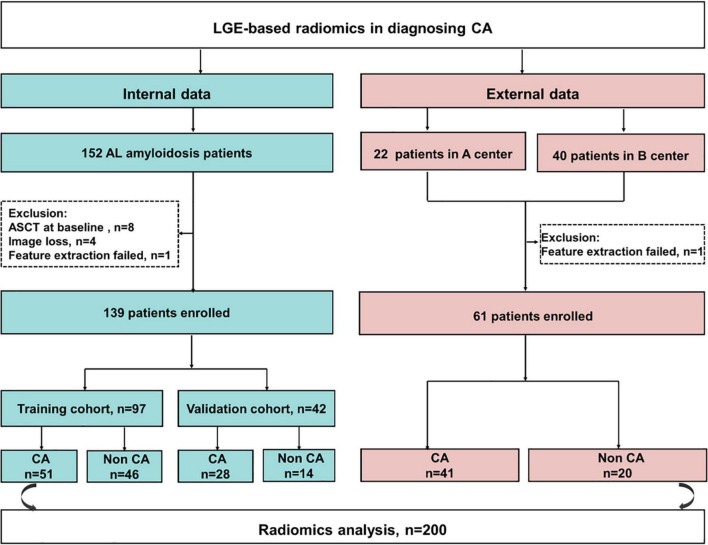
The flowchart of this study. CA, cardiac amyloidosis; AL, light chain amyloidosis; ASCT, autologous stem cell transplant; A center, the Second West China Hospital, Sichuan University, China; B center, Peking Union Medical College Hospital, China.

Of 152 patients from the Jinling Hospital, 13 patients were excluded because of receiving autologous hematopoietic cell transplantation before CMR (*n* = 8), missing MRI images (*n* = 4), and the failure in feature extraction (*n* = 1). Finally, 139 patients were included and were randomly divided into the training cohort and internal validation cohort with the 7:3 ratio. Other 62 patients (40 patients from Peking Union Medical College Hospital, China and 22 patients from the Second West China Hospital, Sichuan University, China) were collected as an external validation set, among whom one patient was excluded due to the failure in feature extraction. Finally, 61 patients were collected in the external validation set (Figure 1). Patients’ demographic, clinical, and CMR characteristics in the training and validation sets are summarized in Table 1. The presence of CA was observed in 79 patients (56.8%) in Jinling Hospital cohort and 41 patients (67.2%) in the external cohort. In the internal cohort, no significant differences between the internal training set and internal validation set regarding clinical characteristics and CMR parameters were noted (all *p* > 0.05). Patients in the external cohort were older (60 vs. 54 years; *p* < 0.001) compared with patients in the internal cohort. Higher levels of troponin [0.066 (0.017–0.163) vs. 0.016 (0.010–0.040) ng/ml, *p* < 0.001] and NT-proBNP [2,423 (308–5,253) vs. 274 (102–1,682) pg/ml, *p* < 0.001] were found in the external validation cohort than in Jinling Hospital cohort. There were 18 (29.5%), 8 (13.1%), 26 (42.6%), and 9 (14.8%) patients in Mayo stages I, II, III, and IV in the external cohort, respectively, while 78 (56.1%), 29 (20.9%), 26 (18.7%), and 6 (4.3%) were in the internal cohort. More than half of the patients were at Mayo stages I and II, reflecting the early stage of CA in the majority of patients in our study. Subjects in the external cohort showed a lower left ventricular ejection fraction (LVEF) (51% vs. 64%, *p* < 0.001), higher left ventricular end-diastolic volume (LVEDV) (126 vs. 100 ml, *p* < 0.001), and higher Left ventricle end-systolic volume (LVESV) (54 vs. 36 ml, *p* < 0.001) compared to those in the internal cohort.

**TABLE 1:** Clinical characteristics and cardiac magnetic resonance (CMR) data in the training and validation data sets.

Variables	Internal cohort (*n* = 139				External cohort (*n* = 61)	
		
	All (*n* = 139)	Training set (*n* = 97)	Validation set (*n* = 42)	*P* value	Validation set (*n* = 61)	*P* value
**Characteristics**						
Male, *n* (%)	75 (53.9)	54 (55.7)	21 (50.0)	0.538	42 (68.9)	
Age, years	53.8 ± 8.4	52.9 ± 8.4	55.7 ± 8.1	0.077	60 ± 9.2	<0.001
**Cardiovascular risk factors, *n* (%)**						
Hypertension	25 (18)	13 (13.4)	12 (28.6)	0.032	5 (8.2)	0.074
Hyperlipidemia	45 (32.4)	32 (33.0)	13 (31.0)	0.814	7 (11.5)	0.008
Diabetes mellitus	10 (7.2)	8 (8.3)	2 (4.8)	0.465	7 (11.5)	0.535
Coronary disease	3 (2.2)	2 (2.1)	1 (2.4)	0.905	0	0.248
**Myocardial enzymes**						
Troponin T, ng/mL	0.02 (0.01, 0.04)	0.02 (0.01, 0.04)	0.02 (0.01, 0.03)	0.801	0.066 (0.017, 0.163)	<0.001
NT-proBNP, pg/mL	274 (102, 1,682)	227 (95.7, 1,645)	543 (121, 1,438)	0.491	2,423 (308, 5,253)	<0.001
Positive CA, *n* (%)	79 (56.8)	51 (52.6)	28 (66.7)	0.124	41 (67.2)	0.168
Mayo stage (I/II/III/IV)	78/29/26/6	50/21/17/4	23/8/9/2	0.129	18/8/26/9	<0.001
**Cardiac MR**						
LVEF, %	63.9 (55.5, 70.0)	64.5 (55.6, 70.2)	64.5 (55.1, 70.1)	0.984	50.8 ± 12.4	<0.001
LVEDV, ml	99.7 (87.2, 117.3)	99.5 (87.5, 117)	101 (88.7, 117)	0.489	123 (106, 149)	<0.001
LVESV, ml	36.1 (26.8, 46.5)	35.8 (26.7, 46.3)	37.2 (27.3, 49.4)	0.785	54.0 (45.8, 79.3)	<0.001
**LGE**						
Total enhanced volume, ml	9.1 (1.8, 25.0)	8.7 (1.93, 20.0)	15.0 (1.81, 40.9)	0.236	5.6 (1.8, 19.1)	0.209
Total enhanced mass, ml	9.6 (1.9, 26.3)	9.1 (2.0, 21.0)	15.8 (1.9, 42.9)	0.236	5.9 (1.9, 20.1)	0.209
LGE extent, %	15.9 (4.7, 36.6)	16.6 (4.9, 37.9)	14.3 (2.5, 36.5)	0.288	9.8 (2.7, 26.6)	0.041

*Data given as mean ± standard deviation (SD), n (%), or median (interquartile range).*

*NT-proBNP, N-terminal pro-B-type natriuretic peptide; LVEF, left ventricle ejection fraction; LVEDV, left ventricular end-diastolic volume; LVESV, left ventricle end-systolic volume; LGE, late gadolinium enhancement.*

### Radiomics Feature Selection and Construction

**FIGURE 2:**
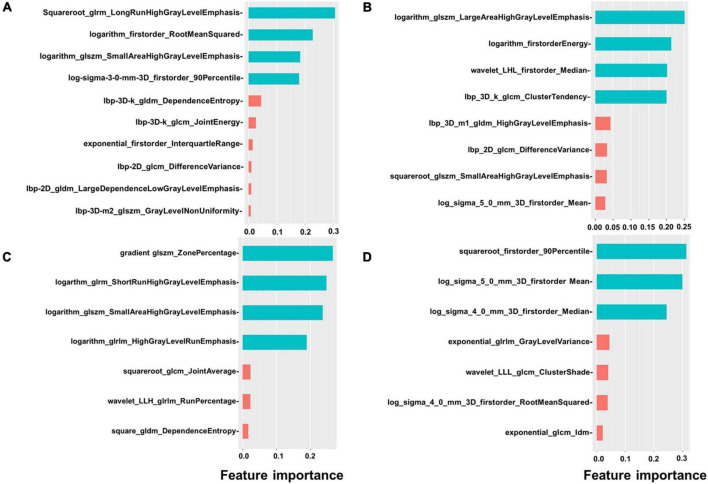
Feature importance of radiomics. The figure shows the feature importance of global **(A)**, basal **(B)**, mid **(C)**, and apical **(D)** radiomics signature. Different colors represent different clusters.

**FIGURE 3:**
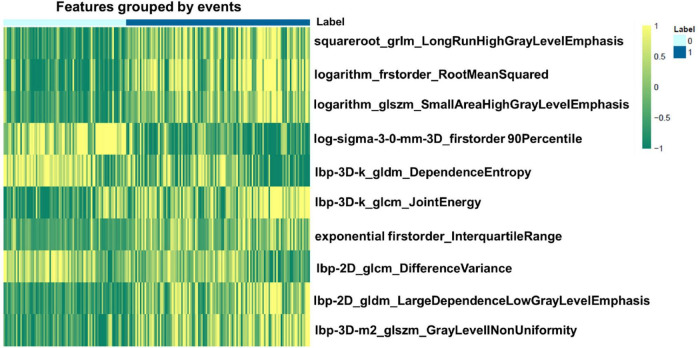
Heatmap of the 10 most relevant radiomics features of global myocardium for differentiating cardiac amyloidosis (CA) from non-CA patients. 0 represents the patients without CA, while 1 represents positive CA patients.

**FIGURE 4:**
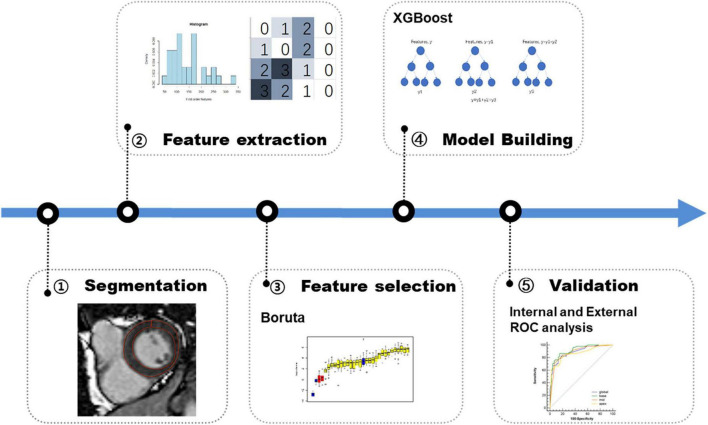
Workflow of the development and validation of radiomics model. First, lesions are manually segmented on late gadolinium enhancement (LGE) MR images for radiomics analysis. Second, a total of 1,906 radiomics features are extracted on global myocardium and three different sections (base, mid-cavity, and apex), respectively. Third, in the training phase, Boruta algorithm is used for feature selection. Fourth, XGBoost machine learning algorithm is used for model building. Finally, in the validation phase, the model is tested in the internal data set and external data sets.

We extracted 1,906 radiomics features from the global, basal, mid, and apical segments of MRI images. A gradient boosting machine (GBM) model was built for the binary classification of patients based on the clinical and imaging features extracted from the CMR images. For feature selection and signature construction, we selected 1,280 high reproducible features of the global myocardium, and 1,174, 270, 93 features of basal, middle, and apical myocardium with the ICCs exceeding 0.80. Boruta-based feature selection was used to sort the importance of the features. Finally, 10, 8, 7, and 7 important features were selected from the 1,906 radiomics features of the full, basal, middle, and apical LV myocardium on CMR images, respectively. Feature importance for radiomics signature is shown in Figure 2. Associations between the 10 global radiomics features and CA are shown in Figure 3. A workflow of the development and testing of the radiomics model is shown in Figure 4.

### Reproducibility of Visual Assessment

The inter-observer reproducibility for a qualitative LGE pattern using ICC was very good [ICC = 0.82 (95% CI: 0.75–0.87)]. Inter-observer agreement was excellent for the semiquantitative QALE score [ICC = 0.92 (95% CI: 0.89–0.94)].

### Radiomics Model Performance

The diagnostic performances and AUC values of the radiomics model in the different sets for the training and validation cohort are shown in Table 2. The radiomics signatures showed favorable discriminating abilities in the training set with AUC values over 0.85 [global: 0.89 (95% CI: 0.83–0.95), base: 0.92 (95% CI: 0.86–0.97), middle: 0.89 (95% CI: 0.82–0.95), and apex: 0.87 (95% CI: 0.80–0.94)]. In the validation cohort, the AUCs were 0.89 (95% CI: 0.78–1.00) for global, 0.89 (95% CI: 0.79–0.99) for base, 0.87 (95% CI:

**TABLE 2:** The performance of radiomics model in the training, internal, and external validation data sets.

	Accuracy (95% CI)	Sensitivity (95% CI)	Specificity (95% CI)	PPV (95% CI)	NPV (95% CI)	AUC (95% CI)
**Global**						
Training	0.78 (0.69–0.86)	0.82 (0.69–0.92)	0.83 (0.69–0.92)	0.84 (0.73–0.91)	0.81 (0.70–0.89)	0.89 (0.83–0.95)
Internal	0.82 (0.73–0.89)	0.96 (0.82–1.00)	0.64 (0.35–0.87)	0.84 (0.73–0.92)	0.90 (0.56–0.99)	0.89 (0.78–1.00)
External	0.81 (0.66–0.91)	0.93 (0.80–0.99)	0.75 (0.51–0.91)	0.88 (0.78–0.94)	0.83 (0.62–0.94)	0.92 (0.85–0.98)
**Base**						
Training	0.86 (0.77–0.92)	0.86 (0.74–0.94)	0.84 (0.71–0.94)	0.86 (0.76–0.93)	0.85 (0.74–0.92)	0.92 (0.86–0.97)
Internal	0.81 (0.66–0.91)	0.79 (0.59–0.92)	0.86 (0.57–0.98)	0.92 (0.75–0.98)	0.67 (0.49–0.81)	0.89 (0.79–0.99)
External	0.87 (0.76–0.94)	0.90 (0.77–0.97)	0.80 (0.56–0.94)	0.90 (0.79–0.96)	0.80 (0.61–0.91)	0.92 (0.85–0.99)
**Mid**						
Training	0.82 (0.73–0.89)	0.84 (0.71–0.93)	0.80 (0.66–0.91)	0.83 (0.72–0.90)	0.82 (0.71–0.90)	0.89 (0.82–0.95)
Internal	0.86 (0.81–0.95)	0.93 (0.77–0.99)	0.71 (0.42–0.92)	0.87 (0.74–0.94)	0.83 (0.56–0.95)	0.87 (0.76–0.98)
External	0.85 (0.74–0.93)	0.90 (0.77–0.97)	0.95 (0.75–1.00)	0.97 (0.85–1.00)	0.83 (0.65–0.92)	0.92 (0.84–0.99)
**Apex**						
Training	0.80 (0.71–0.88)	0.78 (0.65–0.89)	0.83 (0.69–0.92)	0.83 (0.72–0.91)	0.78 (0.67–0.86)	0.87 (0.80–0.94)
Internal	0.81 (0.66–0.91)	0.93 (0.77–0.99)	0.64 (0.35–0.87)	0.84 (0.72–0.91)	0.82 (0.53–0.95)	0.87 (0.74–1.00)
External	0.84 (0.72–0.92)	0.90 (0.77–0.97)	0.70 (0.46–0.88)	0.86 (0.76–0.92)	0.78 (0.57–0.90)	0.90 (0.83–0.98)

*PPV, positive predictive value; NPV, negative predictive value.*

0.76–0.98) for middle, and 0.87 (95% CI: 0.74–1.00) for apex, respectively. In the external validation set, the radiomics approach yielded an AUC of 0.92 (95% CI: 0.85–0.98), 0.92 (95% CI: 0.86–0.99), 0.92 (95% CI: 0.84–0.99), and 0.90 (95% CI: 0.83–0.98) for global and basal, middle, and apical sections, respectively. The DeLong test demonstrated no significant differences between the different sections of LV in the training and validation cohorts (all *p* > 0.05).

### Comparison the Performance of Radiomics With Qualitative, Semiquantitative, and Quantitative Models

**FIGURE 5:**
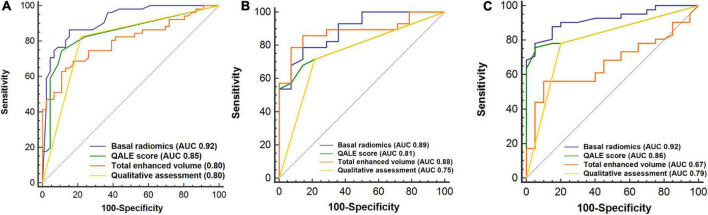
Receiver operating characteristic (ROC) curves analysis for comparison of diagnostic performance of radiomics with qualitative and quantitative LGE assessment. Basal radiomics vs. qualitative (LGE pattern), semiquantitative (QALE score), and quantitative (based on cardiac magnetic resonance (CMR) software) assessment parameters in the training **(A)**, internal **(B)**, and external validation **(C)** cohorts. QALE, query amyloid late enhancement.

We analyzed and compared the performance of radiomics with qualitative, semiquantitative, and quantitative LGE parameters using AUC in the training, internal, and external validation cohorts. The optimal cutoff value of the QALE score > 1 using Youden index achieved a sensitivity of 74.5% (95% CI, 0.60–0.86) and specificity of 89.1% (95% CI, 0.76–0.96) for detecting CA, while total enhanced volume > 10.98 achieved sensitivity and specificity of 62.8% (95% CI, 0.48–0.76) and 89.1% (95% CI, 0.76–0.96) in the training set. The diagnostic accuracy of radiomics was compared against a qualitative LGE pattern, the semiquantitative QALE score and quantitative LGE measurement on the CMR software, and the corresponding results are shown in Figure 5. The radiomics model in the training cohort showed a higher AUC than qualitative and quantitative LGE assessment parameters in the diagnosis of CA (all *p* < 0.05). Although in internal and external validation cohorts, no significant difference was found between radiomics and some semiquantitative and quantitative parameters, radiomics model all outperformed the qualitative LGE pattern (AUC in the training cohort: 0.92 vs. 0.80, *p* = 0.003; AUC in the internal validation cohort: 0.89 vs. 0.75, *p* = 0.022; and AUC in the external cohort: 0.92 vs. 0.79, *p* = 0.008). The results of comparison in the training and validation cohorts are seen in Table 3.

**TABLE 3:** Diagnostic performance of qualitative, semiquantitative, and quantitative parameters in cardiac amyloidosis (CA).

		Internal data (*n* = 139)				External Data (*n* = 61)	
		
		Training cohort (*n* = 97)		Validation cohort (*n* = 42)		External cohort (*n* = 61)	
			
Variables		AUC (95% CI)	*P* value (vs. Radiomics)	AUC (95% CI)	*P* value (vs. Radiomics)	AUC (95% CI)	*P* value (vs. Radiomics)
Qualitative	LGE (±)	0.80 (0.72–0.88)	0.003	0.75 (0.61–0.89)	0.022	0.79 (0.68–0.90)	0.008
Semiquantitative	QALE score	0.85 (0.77–0.92)	0.073	0.80 (0.69–0.92)	0.144	0.86 (0.78–0.94)	0.123
Quantitative	Total enhanced volume, ml	0.80 (0.71–0.89)	0.009	0.88 (0.78–0.99)	0.885	0.67 (0.53–0.81)	<0.001
	Total enhanced mass, g	0.80 (0.71–0.89)	0.009	0.88 (0.78–0.99)	0.885	0.67 (0.53–0.81)	<0.001
	Enhanced volume (%)	0.76 (0.66–0.85)	0.002	0.88 (0.77–0.99)	0.872	0.63 (0.49–0.78)	<0.001
Radiomics	Basal Rad score	0.92 (0.86–0.97)		0.89 (0.79–0.99)		0.92 (0.85–0.99)	

*AUC, area under curve; QALE, Query Amyloid Late Enhancement; Rad-score, radiomics score.*

### Relationship Between Rad-Score and Mayo Stage

For all patients, radiomics of LV global, basal myocardium was moderately correlated with Mayo stage (Spearman’s rho = 0.61, 0.62; all *p* < 0.01). There was no significant correlation between middle or apical Rad-score and Mayo stage (all Spearman’s rho < 0.20, *p* > 0.05). Mayo stage was strongly correlated with NT-proBNP (Spearman’s rho = 0.84; *p* < 0.001) and mildly correlated with LVEF (Spearman’s rho = − 0.37; *p* < 0.001), and total enhanced volume (Spearman’s rho = 0.41; *p* < 0.001). In the internal and external cohort, radiomics of global, basal myocardium was also moderately correlated with Mayo stage (internal cohort, Spearman’s rho = 0.60, 0.56 and external cohort, Spearman’s rho = 0.50, 0.60; all *p* < 0.001).

## Discussion

Our study demonstrated that the radiomics model outperformed the qualitative and quantitative LGE assessment models in the diagnosis of CA. More importantly, radiomics was moderately correlated with the severity of cardiac involvement. To the best of our knowledge, this is the first study to demonstrate the promising value of a radiomics-based approach to diagnose CA based on LGE CMR images.

Late gadolinium enhancement CMR is the most well-established method for identifying cardiac involvement in amyloidosis, with sensitivity and specificity approaching 85–90% (25). However, early cardiac involvement is much more difficult to detect. In CA patients with atypical LGE patterns in such as diffuse transmural, more localized, patchy, and subepicardial LGE, CA is relatively difficult to diagnose and is not easily differentiated from other myocardiopathies. Radiomics provides a novel approach to image interpretation, enabling to extract and analyze quantitative information that cannot be assessed by visual inspection from clinical images, leading to the early detection of CA prior to the appearance of a typical LGE pattern. CMR radiomics has the potential to transform our approach to define image phenotypes and improve diagnostic accuracy, treatment decision, and prognostication (16). Some small proof-of-concept studies have demonstrated a superior diagnostic accuracy of the CMR radiomics analysis to conventional reporting (15–19). In a proof-of-concept study, Baessler et al. (17) demonstrated the feasibility of CMR texture analysis (TA) to accurately differentiate myocardial disease states from healthy hearts, and indicated that TA allows for the diagnosis of hypertrophic cardiomyopathy (HCM) with excellent accuracy, representing a potential novel parameter for reflecting alterations of the myocardium at a tissue level. Radiomics has also shown good performance in differentiating different disease states. For example, Neisius et al. (18) examined the diagnostic ability of radiomics TA on quantitative CMR images to differentiate hypertensive heart disease (HHD) from HCM, and demonstrated that the radiomics analysis of native T1 images provided incremental classification accuracy over global T1 mapping. In another study, Baessler et al. (19) demonstrated the superior diagnostic accuracy of radiomics TA applied to T1 and T2 maps in the diagnosis of acute infarcts like myocarditis. Our study found that the radiomics model outperformed the qualitative and quantitative LGE assessment models in the diagnosis of CA, which further confirmed the superiority of radiomics applications. Although there was no statistical difference between the diagnostic performance of radiomics and semiquantitative QALE score, the AUC of radiomics was still higher than that of the QALE score. In this study, cardiac involvement of patients in the external cohort was more severe than an internal cohort, but total enhanced volume, total enhanced mass, and the percentage of enhanced volume in the external cohort were lower than an internal cohort and these quantitative parameters showed a lower AUC than an internal cohort. It may be because the quantitative assessment of LGE was based on the comparison of normal myocardial tissues, and in patients with diffuse myocardial involvement, it is not possible to outline the normal myocardium; therefore, quantitative measurements in patients with severe cardiac involvement were often underestimated. Also, the radiomics diagnostic performance of external data sets in our study was higher than that of internal data sets, which may be due to the more severe cardiac involvement in the external data sets compared to the internal data sets. However, high AUCs were reported in both internal and external cohorts, indicating radiomics model’s robustness is high with a promising potential of wide generalization in diagnosing CA.

In our study, radiomics performance in global and different LV sections (base, mid-cavity, and apex) has similar diagnostic performance in both the internal and external cohorts. This finding did not seem to support the regional variation of LGE distribution in patients with CA, i.e., “relative apical sparing.” A previous study has shown that the total amyloid volume was significantly higher at the base than at the apex (29). Several studies have reported a base-to-apex gradient in terms of amyloid deposition (29–31). Thus, relative apical sparing is a very consistent and characteristic finding in patients with CA, which describes a reduction in longitudinal strain in the basal and mid-myocardial segments, with relative sparing of the LV apex. In our study, the performance of radiomics model at the basal, mid-ventricular, and apical section showed a slight gradient difference (the AUC is 0.92, 0.89, and 0.87 in the training cohort, respectively), but there is no significantly statistical difference. It is speculated that the LGE in patients with CA is related to the deposition of amyloid fibrils, while radiomics can detect subtle changes such as signal intensity, texture, and shape. Although the amyloid load shows a significant base-to-apex gradient in the typical CA patients, but the different parts of the myocardium have already undergone subtle pathological changes due to CA being a progressive infiltrative disease, which can be detected by radiomics. Nevertheless, the basal radiomics features appear in the optimal section to diagnose CA because it has the highest AUC compared to middle and apical myocardium despite the absence of statistically significant differences. The optimal LV base radiomics analysis can improve the diagnostic accuracy and reduce the overall CMR examination time.

In a very few studies, significant correlations between LGE and clinical, morphological, functional, and biochemical markers have been identified (14, 25). A meta-analysis concluded that the continuum of cardiac involvement in systemic AL and transthyretin amyloidosis (ATTR) amyloidosis with transmural LGE representing advanced CA, and the presence of LGE is associated with increased mortality in patients with systemic amyloidosis and a known or suspected CA (32). Lin et al. found that LGE showed a significant correlation with the New York Heart Association (NYHA) classification, NT-proBNP, and Mayo stage (12). In another study to evaluate the prognostic value of CMR LGE for determining all-cause mortality in AL amyloidosis, the global LGE pattern group showed a higher biomarker stage, and diffuse LGE provided an incremental prognosis over the cardiac biomarker stage in patients with AL CA (33). In our study, we did find a moderate association between global, basal radiomics, and Mayo stage, which suggested that radiomics can reflect the severity of cardiac involvement to some extent. Although radiomics performance in global and different LV sections (base, mid-cavity, and apex) had similar diagnostic performance, but there was no significant correlation between middle or apical Rad-score and Mayo stage in our study, which may be due to a small sample size. A determination is made whether middle and apical radiomics features correlate with the severity of cardiac involvement, and further validation is needed in future studies with larger samples.

### Study Limitations

The present study has several limitations. First, it is a retrospective analysis of prospective internal cohort and retrospective external cohorts. Second, CA was not histopathologically confirmed, and the reference diagnosis was based on the currently available recommendations of the expert consensus (21, 22). Third, the sample size was relatively small, and it is still necessary to increase the sample size in a future study. Fourth, all of our subjects included in this study were AL amyloidosis, thus it cannot extrapolate to ATTR or other myocardiopathies. Fifth, the study focused only on binary diagnosis classification of patients (negative or positive for CA). The role of radiomics in predicting the prognosis of CA merits further studies. Sixth, the threshold of 5 SD was used in the LGE analysis in our study according to previous reports (28, 31). Finally, the LGE technique is limited by the need for intravenous administration of gadolinium-based contrast agents, which were used at a fixed dose during the scan. The radiomics analysis should be explored using other non-contrast enhanced approaches, and this diagnosis performance should also be compared with the other CMR techniques, including T1 mapping.

## Conclusion

In conclusion, the MRI-based radiomics approach is a useful and complementary tool for the detection of CA when diagnostic LGE images are available and have high performance outperforming visual assessment and quantitative CMR parameters. Radiomics is moderately associated with biomarkers of prognosis in CA. Further studies are needed to validate the potential value of radiomics in larger populations and other advanced CMR sequences.

## Data Availability Statement

The original contributions presented in the study are included in the article/Supplementary Material, further inquiries can be directed to the corresponding author/s.

## Ethics Statement

The studies involving human participants were reviewed and approved by Ethics committee affiliation to Jinling Hospital. The patients/participants provided their written informed consent to participate in this study. Written informed consent was obtained from the individual(s) for the publication of any potentially identifiable images or data included in this article.

## Author Contributions

GFY, LJZ, XYZ, and CXT contributed to conception and design of the study. XYZ, CXT, and YKG had contributed to this work in patients’ recruitment, image measurement, and data analysis. XYZ wrote the manuscript. XWT performed the statistical analysis and image processing. SL, JHL, and WWH performed data analysis and interpretation. WCC, JZG, GSR, YKG, YNW, and XL participated with patients’ recruitment. XYZ, CXT, and LJZ were in charge of the drafting of the manuscript or revising it critically for important intellectual content. GML, XHH, YNW, and GFY reviewed and revised the manuscript. All authors approved the final manuscript submitted.

## Conflict of Interest

XWT was employed by Bayer Healthcare. The remaining authors declare that the research was conducted in the absence of any commercial or financial relationships that could be construed as a potential conflict of interest.

## Publisher’s Note

All claims expressed in this article are solely those of the authors and do not necessarily represent those of their affiliated organizations, or those of the publisher, the editors and the reviewers. Any product that may be evaluated in this article, or claim that may be made by its manufacturer, is not guaranteed or endorsed by the publisher.

## Footnotes

1
https://keyan.deepwise.com

2
https://github.com/Radiomics/pyradiomics

3
www.Rproject.org
