# Supplementary material for: Diagnosis of Cardiac Amyloidosis Using a Radiomics Approach Applied to Late Gadolinium-Enhanced Cardiac Magnetic Resonance Images: A Retrospective, Multicohort, Diagnostic Study
Source: Front Cardiovasc Med. 2022 Mar 30;9:818957. doi: 10.3389/fcvm.2022.818957 (PMC9005767; doi:10.3389/fcvm.2022.818957)
Supplement: Supplementary file 1 [file Data_Sheet_1.pdf]

Supplemental Appendix

CMR imaging

Acquisition protocols

The Second West China Hospital, Sichuan University, Chengdu, China

The cine images were acquired with an ECG-gated 2D balanced steady-state free precession (bSSFP) sequence during multiple breath holds. The key parameters were as follows: repetition time (TR)/echo time (TE), 3.3/1.43 ms; flip angle (FA), 55°–70°; voxel size, 1.6 mm× 1.6 mm × 6.0 mm; temporal resolution, 45.6 ms; bandwidth,962 Hz/pixel. Late gadolinium enhancement (LGE) images were collected by a 2D phase-sensitive inversion-recovery (PSIR) gradient-echo pulse sequence with breath-hold. Parameters of the sequence were as follows: TR/TE/FA, 5.2 ms/1.96 ms/20°; voxel size, 1.4 mm × 1.4 mm × 8.0 mm.

Peking Union Medical College Hospital, Beijing, China

Cine images were obtained using balanced steady-state free-precession sequences in consecutive short axis covering the LV. Scanning parameters were as follows: TR, 39.34 ms and TE 1.22 ms; FA, 38°; field of view, 284 mm × 399 mm; matrix size 139 × 208 mm; and slice thickness, 8 mm. LGE imaging was acquired 10–15 min after contrast injection by using an inversion recovery TrueFISP sequence (TR/TE = 870.4/1.18 ms; FA = 40°; slice thickness = 8 mm; field of view = 400 mm× 275 mm; matrix size = 176 × 256.
